# B- and T-Cell Subset Abnormalities in Monogenic Common Variable Immunodeficiency

**DOI:** 10.3389/fimmu.2022.912826

**Published:** 2022-06-15

**Authors:** Saba Fekrvand, Shaghayegh Khanmohammadi, Hassan Abolhassani, Reza Yazdani

**Affiliations:** ^1^ Research Center for Immunodeficiencies, Children’s Medical Center, Tehran University of Medical Science, Tehran, Iran; ^2^ Division of Clinical Immunology, Department of Biosciences and Nutrition, Karolinska Institute, Stockholm, Sweden; ^3^ Division of Clinical Immunology, Department of Laboratory Medicine, Karolinska Institute at Karolinska University Hospital Huddinge, Stockholm, Sweden; ^4^ Primary Immunodeficiency Diseases Network (PIDNet), Universal Scientific Education and Research Network (USERN), Tehran, Iran; ^5^ Department of Neurology, Thomas Jefferson University, Philadelphia, PA, United States

**Keywords:** inborn errors of immunity, primary immunodeficiency, common variable immunodeficiency disorder, CVID, monogenic disorders, B cell subsets, T cell subsets

## Abstract

Common variable immunodeficiency (CVID) is a heterogeneous group of inborn errors of immunity characterized by reduced serum concentrations of different immunoglobulin isotypes. CVID is the most prevalent symptomatic antibody deficiency with a broad range of infectious and non-infectious clinical manifestations. Various genetic and immunological defects are known to be involved in the pathogenesis of CVID. Monogenic defects account for the pathogenesis of about 20-50% of CVID patients, while a variety of cases do not have a defined genetic background. Deficiencies in molecules of B cell receptor signaling or other pathways involving B-cell development, activation, and proliferation could be associated with monogenetic defects of CVID. Genetic defects damping different B cell developmental stages can alter B- and even other lymphocytes’ differentiation and might be involved in the clinical and immunologic presentations of the disorder. Reports concerning T and B cell abnormalities have been published in CVID patients, but such comprehensive data on monogenic CVID patients is few and no review article exists to describe the abrogation of lymphocyte subsets in these disorders. Hence, we aimed to review the role of altered B- and T-cell differentiation in the pathogenesis of CVID patients with monogenic defects.

## Introduction

Common variable immunodeficiency disorder (CVID) is the most prevalent symptomatic inborn error of immunity (IEI) or primary immunodeficiency characterized by hypogammaglobulinemia, impaired production of specific immunoglobulins (Igs) after vaccination and increased susceptibility to infections (1). CVID is a complex and heterogeneous disease and the affected patients present a wide spectrum of infectious and non-infectious clinical manifestations including recurrent bacterial infections of various sites of the body particularly the respiratory tract, inflammatory complications, autoimmunity, interstitial lung disease, enteropathy, granulomatous disease, atopic diseases, lymphoproliferation and malignancy (1, 2).

In the majority of CVID patients, the exact pathogenesis remains unknown and no cause for the immune defect has been detected. In the past two decades, studies have revealed monogenic causes leading to the CVID phenotype, however, these monogenic causes account for the pathogenesis of almost 20-50% of the CVID cases (3). The clinical diagnosis of CVID is an umbrella terminology covering several genetic defects, hence monogenic disorders are now considered to be distinct IEI entities.

Known genetic defects in CVID patients commonly involve maturation, activation and survival of B cells. Hence, mutations in genes encoding the receptors, activators and co-stimulators involved in signaling, activation, survival, migration and maturation of other lymphocytes, particularly T cells, are associated with various degrees of impairment (4, 5). Based on the mutated gene and affected molecule in the B- and T-cell developmental pathways, immunological phenotypes vary between patients with monogenic CVID and this could be helpful in the diagnosis of these diseases.

Regarding identification of B and T cell subsets in CVID patients, we provided an overview on application of flow cytometry in primary antibody disorders (PAD) (including CVID patients) described correct gating strategies along with presenting some flow cytometry examples of CVID patients to guide the researchers for better identifying these cells (6).

**Figure 1:**
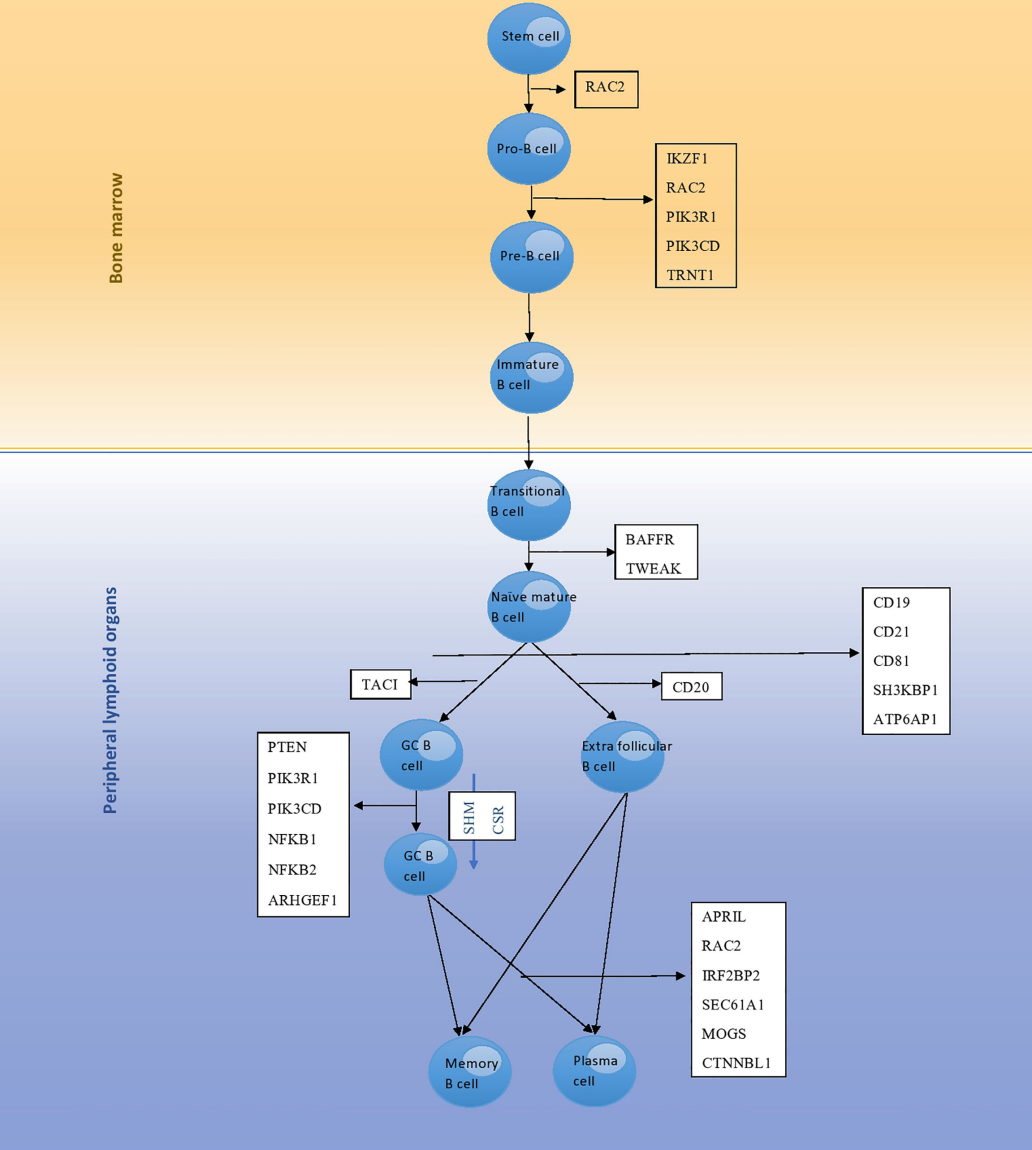
The development pathway of B cells and genes involved in each stage; mutation of these genes leads to monogenic CVID. *RAC2* encodes a protein that is involved in the differentiation of stem cells to pro-B cells, as well as pro-B cells to pre-B cells. Differentiation of pro-B cells to pre-B cells is further regulated by proteins encoded by the *IKZF1*, *PIK3R1*, *PIK3CD* and *TRNT1* genes. *BAFFR* and *TWEAK* encode proteins involved in differentiation of transitional B cells to naϊve mature B cells. Then, naϊve mature B cells undergo selection and enter peripheral lymphoid tissues that is regulated by proteins encoded by the *CD19*, *CD21*, *CD81*, *SH3KBP1* and *ATP6AP1* genes. *TACI* encodes a protein involved in conversion of naϊve mature B cells to germinal center B cells. *CD20* encodes a protein involved in conversion of naϊve mature B cells to extra follicular B cells. Finally, plasma cell differentiation is regulated by proteins encoded by the *APRIL*, *RAC2*, *IRF2BP2*, *SEC61A1*, *MOGS* and *CTNNBL1* genes.

**Figure 2:**
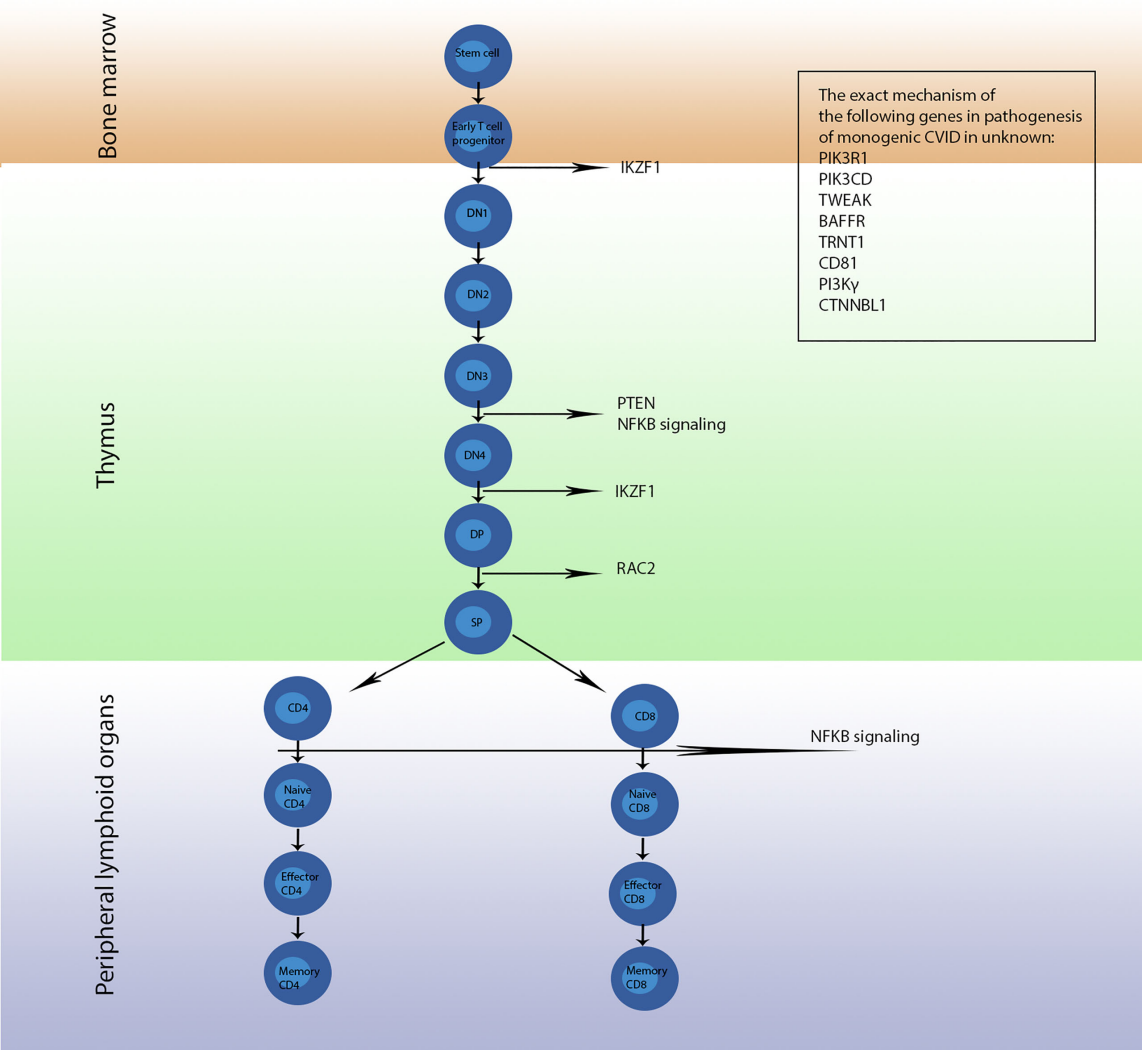
The development pathway of T cells and genes involved in each stage; mutation of these genes leads to monogenic CVID. Conversion of early T cell progenitor to double negative 1 (DN1) cells is regulated by the protein encoded by IKAROS, a transcription factor encoded by the *IKZF1* gene, which later is involved in conversion of stage double negative 4 (DN4) to stage double positive (DP). *PTEN*, *NFKB1* and *NFKB2* encode proteins involved in development of stage DN3 to stage DN4. Then, RAC2 regulates differentiation of DP cells to single positive (SP) cells. Furthermore, NF-κB1 and NF-κB2 regulate the conversion of CD4^+^ cells to naϊve CD4^+^ cells as well as CD8^+^ cells to naϊve CD8^+^ cells. The exact mechanism and location of action of proteins encoded by the *PIK3R1*, *PIK3CD*, *TWEAK*, *BAFFR*, *TRNT1*, *CD81*, *PI3Kγ* and *CTNNBL1* genes are not understood and further studies are required to come to a definite conclusion.

Herein, we aimed to describe an updated review on the impact of monogenic defects in alterations of B- and T-cell subsets and their role in the pathogenesis of monogenic disorders associated with the CVID phenotype. The affected genes and molecules in these monogenic forms of CVID, their role and mechanism of action in the B- and T-cell developmental pathway and the subsequent abnormalities in B- and T-cell subsets will be discussed in detail in the following sections. **Tables 1**, **2** summarize the main B- and T-cell abnormalities observed in the majority of reported patients with monogenic CVID. Also, the B- and T-cell developmental pathways with genes involved in these processes are depicted in **Figures 1**, **2**.

**Table 1:** B-cell subset abnormalities in monogenic CVID.

Genetic defects	Stem cell	Pre-pro-B	Pro-B	Pre-B	Immature B	Transitional B	Mature B	Class-switched memory B cell	Memory B	Plasma cell	Others	Ref.
PI3KCD Gain-of-function APDS1				↑	↑	↑	↓	↓	↓			(7, 8)
PI3KR1 deficiency APDS2						↑	↓	↓	↓			(7, 9)
PTEN deficiency								↓	↓			(10)
CD19 deficiency								↓ / normal	↓			(11–13)
CD81 deficiency						↓			↓	↓		(14)
CD20 deficiency								↓	↓			(15)
CD21 deficiency							↑	↓	↓		↑CD21^low^	(16–18)
TACI deficiency							↓ / normal	↓	↓		↓ marginal zone B cell	(19–21)
BAFF-R deficiency						↑	↓	↓			↓ marginal zone B cell	(22, 23)
TWEAK deficiency						↓	↓	↓	↓			(24)
APRIL deficiency								↓		↓	↑ marginal zone B cell	(25)
TRNT1 deficiency								↓	↓			(26, 27)
NFKB1 deficiency								↓			↓marginal zone B cell	(28)
NFKB2 deficiency								↓			↓marginal zone B cell	(29)
IKAROS deficiency			↓	↓			↓ / normal		↓ / normal			(30, 31)
IRF2BP2 deficiency								↓			↓B cell plasmablasts	(32)
ATP6AP1 deficiency							normal / ↑	↓	↓			(33)
ARHGEF1 deficiency						↑			↓		↓marginal zone B cell	(34)
SH3KBP1 deficiency								↓	↓		↓mature naϊve follicular B cells	(35)
SEC61A1 deficiency										↓		(36)
RAC2 deficiency							↓ / normal	↓	↓			(37)
MOGS deficiency					↑	↑					CD19^+^IgG^+^↓ CD19^+^IgA^+^↓	(38)
PI3Kγ deficiency									↓		↓monocyte ↑eosinophil	(39)
CTNNBL1 deficiency								↓				(40)

APDS, activated phosphoinositide 3-kinase delta syndrome; PTEN, phosphatase and tensin homolog; CD, cluster of differentiation; TACI, transmembrane activator and calcium-modulating cyclophilin ligand interactor; BAFF-R, B cell activating receptor; TWEAK, TNF-like weak inducer of apoptosis; APRIL, a proliferation-inducing ligand; TRNT1, transfer RNA nucleotidyl transferase, CCA-adding 1; NFKB, nuclear factor-kappa-B; IRF2BP2, interferon regulatory factor 2-binding protein 2; ATP6AP1, ATPase H+ transporting lysosomal accessory protein 1; ARHGEF1, Rho guanine nucleotide exchange factor 1; SH3KBP1, SH3-domain kinase-binding protein 1; SEC61A1, Sec61 translocon alpha 1 subunit; RAC2, Ras-related C3 botulinum toxin substrate 2; MOGS, mannosyl-oligosaccharide glucosidase; CTNNBL1, beta-catenin-like protein 1; ↓, decreased; ↑, increased; Ref, reference.

**Table 2:** T-cell subset abnormalities in monogenic CVID.

Genetic defects	Stem cell	Early T cell progenitor	DN1	DN2	DN3	DN4	DP	CD4	Naϊve CD4	Effector CD4	Memory CD4	CD8	Naϊve CD8	Effector CD8	Memory CD8	Others	Ref.
PI3KCD Gain-of-function APDS1									↓					↑	↑		(7, 8)
PI3KR1 deficiency APDS2									↓				↓	↑	↑		(7, 9)
PTEN deficiency								↓								↓NK cell	(10)
CD19 deficiency																	(11–13)
CD81 deficiency																	(14)
CD20 deficiency																	(15)
CD21 deficiency																↑T_EM_	(16)
TACI deficiency																	(19–21)
BAFF-R deficiency																↑NK cells	(23)
TWEAK deficiency						↑						↑					(24)
APRIL deficiency																	(25)
TRNT1 deficiency										↑	↑	↑				↓T follicular helper ↑γδ T	(26)
NFKB1 deficiency																↓regulatory T cells	(28)
NFKB2 deficiency																↓T follicular helper ↓ NK function	(29)
IKAROS deficiency								↓ / normal / ↑				normal / ↑				↓regulatory T cells	(30, 31)
IRF2BP2 deficiency																	(32)
ATP6AP1 deficiency																	(33)
ARHGEF1 deficiency																↓ Myeloid cell	(34)
SH3KBP1 deficiency																	(35)
SEC61A1 deficiency																	(36)
RAC2 deficiency									↓				↓			↓regulatory T cells	(37)
MOGS deficiency																↓ T cell lymphoproliferative test	(38)
PI3Kγ deficiency								↓				↓			Central and effector↓	↓NK cell, ↓TEMRA, ↓regulatory T cells, ↓CD3+ T cell, ↑DNαβT cells, ↑CD4+HLA-DR+ T cells	(39, 41)
CTNNBL1 deficiency																↓ regulatory T cells ↑ T follicular helper-like T cells	(40)

APDS, activated phosphoinositide 3-kinase delta syndrome; PTEN, phosphatase and tensin homolog; CD, cluster of differentiation; TACI, transmembrane activator and calcium-modulating cyclophilin ligand interactor; BAFF-R, B cell activating receptor; TWEAK, TNF-like weak inducer of apoptosis; APRIL, a proliferation-inducing ligand; TRNT1, transfer RNA nucleotidyl transferase, CCA-adding 1; NFKB, nuclear factor-kappa-B; IRF2BP2, interferon regulatory factor 2-binding protein 2; ATP6AP1, ATPase H+ transporting lysosomal accessory protein 1; ARHGEF1, Rho guanine nucleotide exchange factor 1; SH3KBP1, SH3-domain kinase-binding protein 1; SEC61A1, Sec61 translocon alpha 1 subunit; RAC2, Ras-related C3 botulinum toxin substrate 2; MOGS, mannosyl-oligosaccharide glucosidase; CTNNBL1, beta-catenin-like protein 1; ↓, decreased; ↑, increased; DN, double negative; DP, double positive; Ref, reference.

## Activated Phosphoinositide 3-Kinase Delta Syndrome

Phosphoinositide-3-kinases (PI3Ks) are family members of the intracellular downstream signaling transducer enzymes playing a major role in the regulation of cellular functions of immune cells like cell growth, proliferation, trafficking, differentiation and survival through conversion of phosphatidylinositol 3,4-bisphosphate (PIP2) into active phosphatidylinositol 3,4,5-triphosphate (PIP3) upon activation downstream of cytokine receptors, B cell receptors (BCRs), T cell receptors (TCRs) and Toll-like receptors (42). APDS is a recently described IEI caused by heterozygous gain-of-function (GOF) mutations in the *PIK3CD* gene (APDS1) or loss-of-function (34) mutations in the *PIK3R1* gene (APDS2), resulting in increased activity of the PI3Kδ pathway (43). The subsequent overactive PI3K downstream signaling cascade (PI3K/AKT/mTOR signaling pathway) is associated with abnormalities in B cell and T cell differentiation in APDS mimicking CVID phenotype.

Regarding B cell subsets, an increased number of transitional B cells in APDS patients has been demonstrated (7, 44). Given the role of PIK3CD in human B cell development and differentiation in bone marrow (BM), an overt function of PIK3CD could affect B cell maturation and lead to the accumulation of peripheral immature transitional B cells (9, 45, 46). In addition, it has been reported that PI3Kδ hyperactivation provokes the development of CD19^+^B220^−^ cells in mice which are considered equivalent to the expanded transitional B cells in human APDS patients, because both cell types produce interleukin 10 (IL-10). It is deemed that the increase of these cells could be related to the increased susceptibility to *S. pneumonia* infections observed in APDS patients. In addition to transitional B cells, the counts of immature- and pre- B cells have expanded in BM. However, the number of mature circulating B cells has dramatically decreased. These data demonstrate an important role of PIK3CD in early B cell development. On the other hand, increased mature B cells could be due to a compensatory mechanism in response to low B cell numbers. GOF mutations could not only disturb B cell maturation but also disrupt the production of normal memory B cells. Although the levels of IgG and IgA are low in these patients, the IgM level could be normal, indicating that both CVID and hyper IgM phenotypes are expected in APDS. This immunophenotype is more frequent in the APDS2 group than in the APDS1 group (44).

In recent years, we and others investigated the responsiveness of the PI3K pathway by assessing BCR-induced PI3K signaling in CVID and APDS patients. We reported impaired pAkt expression in a manner of BCR-induced PI3K signaling in B-cells of CVID patients (47). On the other hand, GOF in *PIK3CD* or LOF mutations in *PIK3R1* are associated with increased phosphorylation of AKT in B cells of APDS patients (48, 49). Given the similar phenotype of APDS and CVID patients, evaluating the PI3K signaling could also help to delineate some potential differences between them. A new study reported that the presence of GOF mutations in *PIK3CD* does not impair BCR-induced AKT, mTOR and S6 phosphorylation, suggesting a useful confirmatory diagnostic test for APDS in the differential diagnosis from other CVID patients (50).

Concerning T cell abnormalities in APDS patients, it has been reported that more than 60% of the patients demonstrate CD4^+^ T cell deficiency. This reduction is more severe in APDS1 than in APDS2 patients. Among T cell subsets, the profound deficiency primarily affects naϊve CD4^+^ T cells. However, this scenario is converse in CD8^+^ T cells, and a significant increase is observed in activated and effector memory CD8^+^ T cells in APDS patients. Although APDS disorders are categorized as predominantly antibody deficiencies (51, 52), abnormality in T cell subsets arises some similarities with combined immunodeficiency disorders. Affecting both B and T cells subsets in APDS patients makes sense because the PI3K molecule acts downstream of both BCR and TCR.

## PTEN Deficiency

*Phosphatase and tensin homolog (PTEN)* is a tumor suppressor gene encoding PTEN (10). PTEN down-regulates PI3K/AKT signaling by activation of the forkhead box transcription factor (FOXO1). Activation of FOXO1 by PTEN increases IgD BCR expression leading to the production of mature B cells and responsiveness to multivalent antigens. The germinal center is the location of somatic hypermutation (SHM) and class switch recombination (CSR). To produce high-affinity antibodies of the IgG, IgA, or IgE isotype, the development of GC reaction is necessary. GC reaction is controlled by PI3K and FOXO1. Furthermore, FOXO1 contributes to the localization of B cells to the GC. Considering these processes, PTEN-deficient B cells are unable to produce sufficient levels of IgA and IgG isotypes due to abrogated regulation of PI3K (53). A study on mice showed that B cell-specific PTEN-deficient mice had a low level of IgG and IgA (54). On the other hand, defects in PTEN could be associated with abnormality in the count of memory B cells and the development of the CVID phenotype (10). Analysis of a CVID case with PTEN deficiency showed a decreased number of class-switched memory B cells. Recurrent infections and lymphadenopathy were among the clinical manifestations of the aforementioned patient, which also have been seen previously in the patients with decreased numbers of class-switched memory B cells (10, 55).

Regarding T cells, the PI3K pathway also plays a role in the survival, proliferation, and motility of T cells. Hyperactivated PI3K pathway results from PTEN deficiency, interfering with the normal development of T cells. PTEN-deficient mice showed increased thymic cellularity, autoimmunity, decreased requirement for co-stimulation, and lymphoma. It may also lead to a reduction in functional and mature NK T cells (56).

## CD19 Deficiency

The cluster of differentiation 19 molecule (CD19), also known as B-lymphocyte antigen CD19 is a transmembrane protein exclusively expressed on B lineage cells and is encoded by the *CD19* gene (57). CD19 has an important role in the differentiation and activation of B cells (57). CD19 in conjunction with 3 other proteins -CD21, CD81 and CD225- forms the B cell co-receptor complex, which upon antigen encounter signals synergistically with the B-cell antigen receptor and decreases the threshold for receptor-dependent signaling (57). As a result, patients with mutations in the *CD19* gene and low CD19 levels have a defective response of mature B cells to antigenic stimulation.

Regarding B cell subsets, CD19-deficient patients show a normal number of mature peripheral B lymphocytes, decreased number of CD27^+^IgD^+^ and CD27^+^IgD^-^ memory B cells, defective SHM and CSR, impaired antibody response following vaccination and hypogammaglobulinemia (11, 12). Given the crucial role of CD19 expression in B cell differentiative including the formation of B-1, germinal center, and marginal zone (MZ) B cells as well as promoting the survival of naϊve recirculating B cells, *CD19* mutation is accompanied by the aforementioned B cell subset abnormalities (58).

*In vitro* stimulation of CD19-deficient B cells has shown impaired antigen response with decreased serum IgG and IgA levels and decreased to normal serum IgM levels (11, 12). Moreover, these cells have the potential to undergo antigen-dependent differentiation and produce an antigen-selected B-cell–receptor repertoire, albeit with reduced efficiency (11, 12). Contrary to what is expected, decreased serum IgG1 levels, normal Ig response against protein vaccines and switched memory B cell compartment were reported in a CD19-deficient patient (13).

Considering other lymphocyte subsets, no abnormalities are observed in the number and function of T lymphocytes and NK cells in CD19-deficient patients.

## CD81 Deficiency

The cluster of differentiation 81 (CD81) protein is a member of the tetraspanins family encoded by the *CD81* gene. CD81 plays a crucial role in the trafficking and compartmentalization of CD19 receptors on the surface of the B cells. As mentioned above, CD19, CD21, CD81, and CD225, together, form a B cell co-receptor complex, and CD81, after encountering antigens, leads to the assembly of the complex with BCR (59); decreased threshold of antigen needed to induce B cell activation, clonal expansion, and antibody production is the result of this interaction (60). Impaired activation of B cells upon stimulation, decreased CD19 expression, impaired and reduced memory B cell, reduced transitional B cells, reduced plasma cell formation is seen in patients with CD81 deficiency leading to the development of hypogammaglobulinemia (decreased IgG, normal IgM, and normal to low IgA) and CVID (14). The affected patient may manifest symptoms of inflammatory reactions and autoimmunity like thrombocytopenia associated with antiplatelet antibodies. It seems that the CD19 complex has a role in removing autoreactive B cell receptors; thus, CD19-deficient and CD81-deficient patients could have a higher frequency of autoreactive B cells than the normal population (14).

Concerning T cells, CD81 is associated with CD4 and CD8 and co-engages with CD3 (T cell co-receptor), leading to the T cell activation and differentiation to T helper (Th) 2 cells (14). It seems that the association of immature thymocytes and CD81 on stromal cells is required to induce early T cell development (61). CD81 mainly co-stimulates primary human naïve T cells. CD81-deficient B cells are unable to induce a strong T cell response (14). Nevertheless, the lack of CD81 on T cells does not alter the proliferation or T cell response to antigen, and insufficient T cell-related responses are due to the impaired T-B cell interaction (14).

## CD20 Deficiency

The cluster of differentiation 20 molecule (CD20) encoded by the *CD20* gene (*MS4A1*) is a membrane-embedded surface molecule expressed on B cells with a pivotal role in transmembrane Ca^2+^ transport required for the B cell activation, proliferation, development, efficient BCR signaling and differentiation of B-cells into plasma cells (62).

To date, only one case of CD20 deficiency has been reported (15). The index case had intact precursor B-cell differentiation in the bone marrow with appropriate serum IgM levels and normal total B-cell counts, but reduced circulating class-switched memory B cells, decreased isotype switched Igs, and low IgG antibody titers (15). *In vitro* assays showed a decreased frequency of somatic hypermutations in IgG heavy chain genes and impaired T cell-independent IgG antibody formation (15). Moreover, an IgG against tetanus toxoid antigens could be produced upon repeated booster immunization (15). In line with the conserved function of CD20 in the generation of T cell-independent antibody responses, CD20-deficient mice had a reduced ability to respond to T cell-independent antigens (15). Also, T-dependent humoral immunity is found to be diminished in CD20-null mice (63).

Regarding T cell subsets, the number of CD3^+^, CD4^+^, CD8^+^ T cells as well as CD16^+^56^+^ NK cells were reported to be within the normal range (15).

## CD21 Deficiency

Complement receptor type 2 (CD21) encoded by the *CR2* gene is a member of the complement receptor protein family that regulates complement activation. C3 fragments (i.e., C3d) are the primary ligands of CD21 (64). Although some references mention the contributing role of C3d in humoral immunity, it seems that it is only accurate in mice and not humans (65). A case of CVID with the absence of CD21 on B cells showed a reduced number of class-switched memory B cells and hypogammaglobulinemia (reduced IgG and IgA) (16). Another patient with CD21 deficiency showed increased naïve mature B cells and decreased memory B cells. Interestingly, the patient showed a close to the normal frequency of SHM in memory B cells (17). Moreover, two other siblings with *CR2* mutation showed decreased levels of class-switched memory B cells and low to normal IgG (18). CD21 plays a role in antigen trapping in B cell follicles (16). As previously mentioned, CD21 is associated with CD81/CD19 complex and plays a role in lowering the threshold needed to induce B cell activation and antigen processing (64, 66). The function of CD21 is not as crucial as the function of CD19 and CD81. An increased signaling threshold may explain the increased memory cells, and it may be restored by increased expression of CD19 (17).

CD21 is required for T-dependent antigen responses (66). It also contributes to antigen presentation by facilizing the internalization of immune complexes by B cells (64). We expect to observe an increased number of CD21^low^ B cells in patients with CD21 deficiency. Also, an increased percentage of CD21^low^ B cells is associated with autoimmunity and splenomegaly (60, 67). Although CD21 has been detected in T cells, its role in T cells is not completely understood (68). The results of a study showed that T effector memory (TEM, CCR7^-^CD45RA^-^) cells were increased in two siblings with CD21 deficiency (18). More studies are required to determine the exact role of CD21 in T cells.

## TACI Deficiency

Transmembrane activator and calcium-modulating cyclophilin ligand interactor (TACI), also known as tumor necrosis factor receptor superfamily member 13B (TNFRSF13B) is encoded by the *TNFRSF13B* gene functioning as a cell membrane receptor mainly expressed on the surface of B-cells and plasma cells (69). The tuned function of TACI results in B-cell differentiation, up-regulation of activation-induced cytidine deaminase (AID), isotype CSR, and Ig production especially in a T-independent manner (19, 69). TACI binds to its ligands “a proliferation-inducing ligand” (APRIL) and “B cell-activating factor” (BAFF) that signal through TACI, and activate several transcription factors including the nuclear factor of activated T cells (NFAT), the activator protein 1 (AP1), and nuclear factor-kappa-B (NF-κB) and finally induce CSR to IgA and IgG in naϊve B cells (70). Thus, TACI deficiency is presumed to lead to reduced numbers of class-switched memory B cells, loss of class switch and ultimately hypogammaglobulinemia.

Regarding B cell subsets, patients with *TACI* mutations show variable B cell phenotype, with normal or low numbers of B cells (19–21). A proportion of patients have low class-switched memory B cell numbers and low marginal zone cells (19–21). During the B cell development, TACI plays an important role in B-1 cell maintenance (71) as well as the B cell terminal differentiation into plasma cells (72) leading to low serum IgG and IgA, normal to low serum IgM based on the type of mutation and mono- or bi-allelic defects of *TACI* (19–21). The affected patients also show poor vaccination responses (19–21). Inappropriate levels of TACI signaling may abolish immune regulation, subsequently inducing autoimmune diseases, which is in agreement with the high frequency of autoimmunity in TACI deficiency along with susceptibility to infections of respiratory and gastrointestinal tracts and lymphoproliferation (73).

Considering T cells, no major abnormalities have been observed in T cell subsets and their proliferative responses in TACI-deficient patients (21).

## BAFF Receptor Deficiency

B cell-activating factor receptor (BAFF-R) belongs to the TNF-receptor superfamily encoded by the *TNFRSF13C* gene. While immature B cells convert to transitional B cells, expression of the BAFF-R inhibits premature cell death by receiving the pro-survival signaling. Warnatz *et al.* reported two siblings with BAFF-R deficiency who developed CVID. They had decreased levels of IgG and IgM but normal IgA. Their cell subset analysis showed B-cell lymphopenia, reduced numbers of class-switched memory B cells, and developmental arrest after the transitional stage and before the cells became mature follicular B cells (22). Another case with BAFF-R deficiency mimicked the symptoms of Good syndrome, but immunologically presented severe hypogammaglobulinemia (decreased IgG and undetectable IgM and IgA), reduced numbers of class-switched memory B cells, and increased NK cells. He had a normal proliferative T cell response (23).

BAFF-R induces the PI3K pathway, which its role in B cell development and proliferation has been previously discussed. Evidence demonstrates that not all the B cell subsets require BAFF signaling to survive or develop. It seems that follicular and marginal zone B cells are highly dependent on BAFF signaling, but B1 B cells (in mice, not humans) and switched memory B cells can survive in the absence of BAFF signaling. Transitional 1 B cells are unable to differentiate into naïve and marginal zone B cells in BAFF-R-deficient patients. Moreover, the presence of IgA^+^ plasma cells and increased IgA serum concentrations after vaccination with T-dependent antigens in patients with BAFF-R deficiency is another evidence of the fact that some B cells still can develop into the plasma cells (74). Antibody response to the T-independent antigens is also impaired in patients with BAFF-R deficiency (60) since IgM^+^ CD27^+^ marginal zone B cells are mandatory for controlling infections with encapsulated bacteria (74).

BAFF-R signaling also plays a role in the survival and activation of T cells *via* the PI3K-AKT signaling pathway (75). Based on an animal study, BAFF can increase CD3^+^CD4^+^, CD4^+^CD25^+^, CD4^+^CD69^+^, and CD4^+^CD154^+^ T cells, and decrease CD4^+^CD62L^+^ T cells (75).

## TWEAK Deficiency

TNF-like weak inducer of apoptosis (TWEAK), also known as TNF ligand superfamily member 12 (TNFSF12) is a cytokine belonging to the TNF ligand family encoded by the *TNFSF12* gene (76). TWEAK is described as having a role in BAFF signaling and B-cell survival (24). Hence, *TNFSF12* mutations are presumed to inhibit BAFF-dependent B-cell survival and proliferation as well as Ig class switching through interaction with BAFF and down-regulation of the non-canonical BAFF-induced NF-κB pathway.

To date, only one family with 3 cases has been reported with TWEAK deficiency (24). Regarding B cells, the affected cases had normal or reduced total B cells, class-switched memory B cells numbers, impaired antibody responses to T-cell-dependent and polysaccharide vaccines, and low levels of IgM, IgA, and IgG or normal levels of IgG with low serum levels of IgG2 and IgG4 subclasses. As mentioned above, BAFF-mediated survival signals are particularly critical during the transition between transitional type 1 (T1) to type 2 (T2) immature B cell stage and loss of BAFF-mediated survival signals such as TWEAK deficiency leads to a block of B cell maturation at the T1 immature B cell stage (77).

Concerning T cell subsets, a slightly increased percentage of TCRαβ^+^CD4^−^CD8^−^ T cells (double-negative T cells), increased number of total T cells (especially CD8^+^ T cells) and normal level of CD4^+^ T cells were observed in these patients (24). Animal studies have revealed the critical role of TWEAK in attenuating the transition from innate to adaptive TH1 immunity (78), highlighting the correlation between TWEAK and CD8^+^ T cell activation. However further studies are required to understand the exact mechanism of TWEAK in the pathogenesis of T cell subset abnormalities observed in TWEAK deficiency.

## APRIL Deficiency

A proliferation-inducing ligand (APRIL), also known as TNF ligand superfamily member 13 (TNFSF13) is a member of the TNF ligand superfamily and is encoded by the *TNFS13* gene (79, 80). APRIL plays a pivotal role in inducing the differentiation of memory B cells to plasma cells and immunoglobulin production. Thus, *TNFSF13* mutations are presumed to be associated with impaired plasma cell differentiation and immunoglobulin production. To date, only one patient with APRIL deficiency has been reported (25). The index case presented with normal total B cells, slightly decreased number of class-switched memory B cells, markedly reduced level of plasma cells, increased circulating marginal zone B cells and hypogammaglobulinemia (25).

As mentioned above, interactions between APRIL and the B cell surface TNF receptor superfamily (strongly BCMA and to a lesser strength TACI) play crucial roles in B cell development, differentiation and terminal maturation (25), thus patients with APRIL deficiency will have difficulty in maintaining the lifelong humoral immunity. In this regard, animal studies have also shown the relation between APRIL and long-term maintenance of memory B cells in animal models (81, 82).

Considering other lymphocyte subsets, no abnormalities were observed in T lymphocytes, NK cells and monocytes of the reported APRIL-deficient case (25). However, an animal study on APRIL-deficient mice revealed increased numbers of CD4^+^ effector/memory T cells (83). Thus, further studies with more APRIL-deficient cases are required to understand the probable role of APRIL in development of other lymphocyte subsets.

## TRNT1 Deficiency

*TRNT1* gene encodes transfer RNA (tRNA) nucleotidyltransferase, CCA-adding 1 (TRNT1) protein adds CCA terminus to the 3-prime end of tRNA precursors. This process is necessary for the participation of tRNAs in protein biosynthesis (26). As seen in several reports, TRNT1 deficiency leads to sideroblastic anemia with B cell immunodeficiency, periodic fever, and developmental delay (SFID syndrome) (27). Immunophenotyping of the patient with *TRNT1* gene mutation showed increased CD8^+^ cells, CD4^+^ terminally differentiated effector memory helper T lymphocytes (CD4^+^ T_EMRA_), and CD4^+^ effector memory lymphocytes. In these patients, T follicular helper cells and switched memory B cells were reduced, and B-cell maturation in bone marrow was blocked at the CD19^+^CD10^+^CD20^+/−^ pre-B-cell stage (26, 84). To the best of our knowledge, the exact mechanism of TRNT1 deficiency leading to the development of CVID is not completely understood, but B cell deficiency may be due to the increased endoplasmic reticulum (ER) stress response (similar to activated T cells) (84). This hypothesis should be investigated in future studies

## NFKB1 Deficiency

NF-κB is a family of transcription factors consisting of 5 subunits with pivotal roles in the development, survival, and activation of B lymphocytes (85). *NFKB1* gene encodes nuclear factor-kappa-B p105 subunit (NF-κB1), and is almost present in all the cell types of the human body. NF-κB1 plays a major role in different pathways, including cell survival, inflammation, cell growth, and stress responses. Together with other subunits, the NF-κB complex regulates the expression of several target genes, such as cytokines, chemokines, growth factors, apoptosis regulators, receptors, etc. (28). Monoallelic mutation in *NFKB1* is the most frequent cause of monogenic CVID. Patients with this defect manifest hypogammaglobulinemia due to decreased switched memory B cells, and diminished lymphocyte proliferation after stimulation with B cell mitogens. It seems that the patients usually have a normal response to T-dependent and T-independent antigens (86).

Maturation of the B cells, T-independent antibody response, B cell survival and differentiation, and CSR signaling pathways are associated with NF-κB signaling. NF-κB protects lymphocyte precursors from apoptosis. The NF-κB pathway is also crucial for the survival of developing B cells in the spleen, and complete deficiencies in its complex lead to arrest at or near T1 B cells to T2 B cells step. As a consequence of the absence or decreased levels of marginal zone B cells can be observed. B1 cells also require NF-κB1 and NF-κB2 for their formation. Even activation and differentiation of follicular B-cells require NF-κB signaling. Additionally, NF-κB plays a role in the survival of activated B cells mainly due to stimulation through BCR (87). Moreover, BAFF-R signaling and TACI signaling activate the canonical NF-κB1 pathway and non-canonical NF-κB2 pathway in B cells, respectively, leading to the B cell development, survival, and activation (28).

Regarding T cells, NF-κB1 plays a role in the activation and proliferation of naïve T cells. Besides, NF-κB controls the expression of cytokines in T cells, survival of T cells, and CD4 Th differentiation *via* antigen-presenting cell-dependent and T-cell intrinsic mechanisms. Differentiation of T helper 2 cells is selectively regulated by NF-κB1 (87). Moreover, NF-κB plays a role in regulatory T cell (Treg) generation since NF-κB1 deficiency leads to abrogated Tregs generation (87).

## NFKB2 Deficiency

The nuclear factor NF-kappa-B p100 subunit (NF-κB2) plays the main role in the non-canonical pathway of NF-κB encoded by the *NFKB2* gene. Patients with *NFKB2* mutation showed reduced numbers of switched memory and marginal zone B cells, hypogammaglobulinemia, and weak antibody response to antigens. Decreased number of T follicular helper cells, impaired NK cell function, and severe B-cell deficiency were also reported in some cases (29).

It seems that the non-canonical NF-κB pathway plays a major role in the B cell maturation and survival in mice (88). Similar to NF-κB1, NF-κB2 deficiency can lead to arrest at or near the T1 to T2 differentiation step and, therefore, the absence of marginal zone and follicular B cells. NF-κB2 deficiency can also diminish B1 cells (87).

## IKAROS Deficiency

DNA-binding protein IKAROS (also known as Ikaros family zinc finger protein 1) is a member of a family of hematopoietic zinc-finger transcription factors encoded by the *IKZF1* gene and is involved in gene expression *via* chromatin remodeling (30). IKAROS is a regulator of immune cells development and differentiation, mainly in early B cells and CD4^+^ T cells. This protein functions in the specification and maturation of the T lymphocyte as well as CD4 versus CD8 lineage differentiation (89–91). This molecule is also required for the development of the earliest B cell progenitors and at later stages of B cell development during VDJ recombination in class switching of the antibody isotypes and B cell receptor expression (92). Thus, mutations in *IKZF1* and dysfunctional IKAROS are presumed to affect the expression of BCR in B cells leading to deregulation of the BCR signaling during B cell development, low proliferation rate and increased apoptosis of the B cells.

IKAROS binds many genes required for BCR signaling, Ig recombination, cell growth and proliferation and acts as an important transcription factor in pre-B cell differentiation (93). Patients with haploinsufficiency of IKAROS deficiency show a low to a normal number of total and memory B cells along with a marked decrease in at least two of the three major Ig isotypes (IgG, IgM, and IgA), demonstrating a defect in IKAROS involving B cell development in the bone marrow. On the other hand, IKAROS controls triggering and isotype selection during CSR by controlling epigenetic marks and the transcription process at constant region gene promoters (92). Thus, the whole B cell development process from pro-B cell to switched memory B cells should be considered in the patients.

Considering T cell subsets, these patients have normal to an increased number of total T cells, low, normal or increased number of CD4^+^ T cells, normal to an increased number of CD8^+^ T cells, reversed CD4/CD8 ratios with increased CD8^+^ T cells, reduced regulatory T-cell counts and normal *in vitro* T cell proliferation responses (30, 31). During the T cell development pathway, IKAROS controls the differentiation of T cells and development by the intensification of pre-TCR and TCR (94). It also functions in regulating negative selection as well as CD4 versus CD8 lineage development in the thymus (95). Of note, dominant-negative mutations of *IKZF1 are* associated with a lack of memory T cell generation and can develop the combined immunodeficiency phenotype.

## IRF2BP2 Deficiency

Interferon regulatory factor 2-binding protein 2 (IRF2BP2) is encoded by the *IRF2BP2* gene and belongs to a family of proteins that regulate transcription of type I interferon (IFN I) and proteins downstream to IFN I and IFN II (32). Mutation in the *IRF2BP2* gene in several patients showed impaired development of B cell plasmablasts. Although the patients had a normal total B cell count, their switched memory B cells were reduced. They also had decreased or undetectable serum levels of IgM, IgA, and IgG2. Autoimmune diseases, such as type I diabetes mellitus, were also present in some of the members of the family with *IRF2BP2* mutation (32).

IRF2BP2 represses the NFAT family by interacting with the C-terminal domain of NFAT1. NFAT family plays a major role in the regulations of cell cycle-, differentiation-, and apoptosis-related genes. The presence of IRF2BP2 decreased the production of interleukin (IL)-2 and IL-4 by CD4^+^ T cells (96). IL-2 induces plasma cell differentiation. Therefore, IRF2BP2 deficiency could impair antibody production and B cell development *via* this mechanism mimicking a CVID phenotype.

## ATP6AP1 Deficiency

ATPase H+ transporting lysosomal accessory protein 1 (ATP6AP1) is encoded by the *ATP6AP1* gene and is an accessory subunit of the vacuolar H^+^ -ATPase protein pump that is required for luminal acidification of secretory vesicles (97). ATP6AP1 deficiency is a subclass of congenital disorders of glycosylation (CDG) and is characterized by N- and O-glycosylation defects manifesting with immunodeficiency, hepatopathy and cognitive impairment (33).

ATP6AP1-deficient patients display hypogammaglobulinemia; also some of the patients are reported to respond very poorly to childhood vaccination (33, 98). Moreover, two patients were reported to have high-normal levels of naϊve B cells and decreased the number of switched memory B cells, suggesting a problem in terminal B-cell differentiation (33).

The exact mechanism of ATP6AP1 in the pathogenesis of CVID is unknown and requires further studies. ATP6AP1 is reported to be involved in B cell differentiation, antigen processing and antibody production *via* acidification, membrane trafficking and fusion events (99–102). Furthermore, several membrane-bound proteins involved in B-cell activation such as CD19 and CD40 are glycosylated, and proper detection of antigens and Ig production require fucosylated IgG-BCR (99). Therefore, a glycosylation defect such that observed in ATP6AP1 deficiency might affect B-cell activation and antibody production. Altogether, mutant *ATP6AP1* could affect B-cell activation and/or function leading to decreased levels of Igs and recurrent infections (33).

To date, no studies have been reported regarding the role of ATP6AP1 in T cell homeostasis and further investigations are required to evaluate the possible T cell subset abnormalities in patients with ATP6AP1 deficiency.

## ARHGEF1 Deficiency

Rho guanine nucleotide exchange factor 1 (ARHGEF1) protein belongs to the guanine nucleotide exchange factor (GEF) family encoded by the *ARHGEF1* gene. ARHGEF1 regulates proteins of the RAS superfamily and RhoA guanosine triphosphatases (GTPase). Lymphocytes with *ARHGEF1* gene mutation showed low RhoA activity and low cortical F-actin polymerization (34). The regulation of actin cytoskeleton dynamics, confining PI3K/AKT signaling, and restricting B lymphocytes and myelocytes are attributed to ARHGEF1 function (34). Patients with mutation in the *ARHGEF1* gene had an increased frequency of transitional B cells, decreased marginal zone and memory B cells, impaired antibody production in response to T-dependent and T-independent antigens, and germinal centers with small sizes (which is associated with impaired function and development of B cells). They also had impaired actin polymerization and, consequently, impaired B and T cell migration. Immature myeloid cells were also present in the blood sample of the patient (34). Inability to suppress AKT activity is another feature of cells with ARHGEF1deficiency (103). The exact role of ARHGEF1 in the pathogenesis of CVID needs more evaluation.

## SH3KBP1 (CIN85) Deficiency

SH3-domain kinase-binding protein 1 (SH3KBP1) also known as CBL-interacting protein, 85-kD (CIN85) is encoded by the *SH3KBP1* gene. This protein is an adaptor protein with multiple functions including vesicle trafficking, cytoskeleton remodeling, and ubiquitinoylation-dependent activation is required for downregulation of BCR signaling (104). Mutant *SH3KBP1* is associated with intrinsic defects in BCR-mediated activation and antibody production. *In vitro* studies have shown specific defects in the canonical activation of NF-κB and up-regulation of NF-κB-mediated activation markers CD86 and intercellular adhesion molecule (ICAM)–1 in response to stimulation of the BCR suggesting the nonredundant functions of CIN85 for humoral immune responses (35).

To date, only two patients with SH3KBP1 deficiency have been reported; these patients displayed normal numbers of B-cell subsets including transitional, naϊve, memory B cells and plasmablasts, but a decrease in IgM^+^IgD^+^ memory, IgM only memory, IgG switched memory and IgA switched memory B cells as well as low serum levels of IgM, IgG (total IgG or some of IgG subclasses) and/or IgA, impaired response to polysaccharide vaccination (35). CIN85 is a key component of the BCR-associated process needed for the initiation and continuation of antigen defection signal transduction. It is also required for normal development or maintenance of the B-1a B cell compartment with mutant CIN85 leading to reduced numbers of mature B cells (105).

Regarding T cell subsets, CD3^+^, CD4^+^, CD8^+^, regulatory and memory CD8^+^ T cells were all reported to be normal in number (35). The physiological function of CIN85 in T cells is still unknown and more studies are required to further evaluate T cell subset abnormalities in SH3KBP1-deficient patients.

## SEC61A1 Deficiency

Sec61 translocon alpha 1 subunit (SEC61A1) is one of the subunits of the heteromeric SEC61 complex encoded by the *SEC61A1* gene. The SEC61 complex is a membrane channel located in the membrane of the ER and facilitates the translocation of proteins across ER membrane. ER development is one of the main steps of differentiation of B cells to plasma cells since they are the main producer of antibodies with high Er stress for rapid production of massive Ig secretion (36). Patients with mutation in the *SEC61A1* gene had low levels of IgG, IgM, and IgA with a lack of response to T-independent antigens. Although they had a normal count of B- and T-cell subpopulations, their B cell could not normally develop into plasma cells. These mutations led to a lower antibody production capacity, decreased plasma cell survival, impaired co-translational protein translocation, and impaired Ca^2+^ homeostasis. *In vitro* stimulation of B cells showed the patients carrying *SEC61A1* mutation have impaired differentiation of peripheral B cells to CD27^+^CD38^high^ plasmablasts and CD_38_^high^CD138^+^ plasma cells (36). This data demonstrates that *SEC61A1* mutation high probably influences B cell subsets with late differentiation such as plasmablasts.

## RAC2 Deficiency

Ras-related C3 botulinum toxin substrate 2 (RAC2) is a GTPase and member of the Rac subfamily of the family “Rho family of GTPase” exclusively expressed on hematopoietic cells and is encoded by the *RAC2* gene (106). Members of the Rho family of GTPases are a group of cellular signaling molecules binding downstream effectors to regulate a diverse array of cellular events, including the control of cell growth, cell cycle, actin cytoskeleton reorganization, gene transcription and the activation of protein kinases (107).

Mutations in the *RAC2* gene have been reported in several patients with various forms of IEI indicating mutations in the same gene may affect the immune system variably. For example, autosomal dominant-negative (34) *RAC2* mutations have been reported in patients with phagocytic immunodeficiency with preferentially affected granulocytes (108–110), while autosomal-dominant activating (GOF) mutations of *RAC2* are reported to lead to combined immunodeficiency (111–113) or severe combined immunodeficiency (SCID) (114) and have a prevalent effect on T lymphocytes. Furthermore, a recent study has reported two patients with the autosomal recessive LOF mutation, who had characteristics of CVID and showed modest alterations in primary and secondary neutrophilic granules associated with chemotactic defects and a milder immunologic phenotype than those with autosomal dominant *RAC2* mutations (37). Considering B cell subsets, the RAC2-deficient patients with CVID phenotype displayed a reduced to a normal percentage of B lymphocytes, reduced serum level of IgG, IgA and IgM and poor response to polysaccharide vaccination (37). *In vivo* animal studies on Rac2^−/−^ mice have revealed peripheral blood B lymphocytosis with marked reductions in B1a and marginal zone B lymphocytes along with hypogammaglobulinemia suggesting a deficiency in BCR-mediated signaling and B cell development in a Rac2-dependent manner (115). Another murine study also suggested a critical role of RAC2 in BCR signal transduction-associated up-regulation of BAFF-R as a key survival molecule required for B cell development and maintenance (116).

Regarding T cell subsets, the RAC2-deficient patients with CVID phenotype show decreased percentages of naϊve CD4^+^ and CD8^+^ T cells, reduced percentage of regulatory T cells and slightly low CD4/CD8 ratio (37). RAC2 plays a critical role during multiple stages of T-cell development by regulating survival and proliferation signals and Rac2-knockout mice show reduced immature CD4^+^CD8^+^ and mature CD4^+^ populations in the thymus as well as CD4^+^ and CD8^+^ populations in the spleen (117).

## Mannosyl-Oligosaccharide Glucosidase Deficiency

*MOGS* gene encoding mannosyl-oligosaccharide glucosidase plays a role in processing N-linked glycoproteins. It particularly cleaves the distal alpha-1,2-linked glucose residue in the Glc_3_Man_9_GlcNAc_2_ oligosaccharide precursor (38). A case report of two patients with *MOGS* mutation showed normal B cells and plasma cells. The patients had abnormal N-glycosylated Igs; *in vivo* studies showed a lower half-life of IgG in comparison with control cells that could be the underlying cause of hypogammaglobulinemia. The patients also had impaired antibody response to viruses with a glycosylated capsule. Interestingly, the patients with *MOGS* mutation had no recurrent or severe bacterial infection, and the underlying reason is not completely understood (38).

## Phosphatidylinositol 3-Kinase-Gamma

PI3Kγ is encoded by *PIK3CG* gene and phosphorylates PtdIns(4,5)P2 (Phosphatidylinositol 4,5-bisphosphate) to generate PIP3 (39). The produced second messenger regulates many signaling cascades related to cell survival, growth, proliferation, and motility, as well as cellular responses to growth factors, cytokines, chemokines, and antigen receptor stimulation (39). Although PI3Kγ is mainly known for its roles in myeloid cells, knockout (KO) mice show impaired antibody response and T cells. PI3Kγ is also involved in TLR-related pathways (39). While T cells activate *via* PI3Kγ and PI3Kδ, B cells mainly respond through PI3Kδ; therefore, we expect more T cell defects than B cell defects in PI3Kγ-deficient patients (41). A patient with mutated PI3Kγ showed pathological accumulation of T cells, autoimmune cytopenia, hypogammaglobulinemia, thrombocytopenia, lymphopenias, eosinophilia, lymphadenopathy, and splenomegaly. She had decreased counts of CD8^+^ T cells, CD8^+^ central memory, CD8^+^ effector memory, CD8^+^ T_EMRA_, CD4^+^CD25^+^ T cells, NK cells, monocytes, and memory B cells, and increased levels of DNαβT cells, CD4^+^HLA-DR^+^ T cells, and eosinophils. It seems that the patients with PI3Kγ deficiency have impaired response to TCR stimulation (39). Another patient with PI3Kγ deficiency had thrombocytopenia, decreased number of lymphocytes, CD3^+^ T cells, CD4^+^ T cells, and NK cells (41).

## CTNNBL1 Deficiency

Beta-catenin-like protein 1 (CTNNBL1) is encoded by the *CTNNBL1* gene and is a component of the pre-mRNA processing factor 19 (Prp19)- cell division cycle 5–like (CDC5L) complex that forms an integral part of the spliceosome and is required for activating pre-mRNA splicing (40). Furthermore, CTNNBL1 is identified as an AID-interacting protein through which it participates in SHM as well as CSR (118). Thus, mutations in *CTNNBL1* and regions in *AID* required for CTNNBL1 interaction is presumed to yield diminished Ig hypermutation and class switching (118).

Approximately 20% of CVID patients develop autoimmune cytopenias (16) following defective early B cell development and the resultant autoreactive B cells that secret autoantibodies targeting erythrocytes and/or platelets (1). Recently, a CVID patient with autoimmune cytopenias (CVID+AIC) was found to harbor a rare mutation in the *CTNNBL1* gene (40). Regarding B cell subsets, the patient showed decreased class-switched memory B cells and hypogammaglobulinemia (40).

Considering T cell subsets, a decreased frequency of Tregs as well as increased circulating T follicular helper–like (Tfh-like) T cells were observed (40). However, further studies are required to understand the possible role of CTNNBL1 in T lymphocyte development pathway.

## B- and T-Cell Abnormalities in Non-Genetic Forms of CVID

B- and T-cell subsets are also affected in non-genetic form of CVID. We demonstrated a reduced levels of CD4^+^ T cells, Tregs, and Th17 in one study on patients with CVID without known monogenic disease; while the frequencies of Th1, Th1-like Th17, and Th22 subsets were normal. It seems that infection and the lymphoproliferative phenotype of the patients are associated with the imbalance of Th17 and Tregs (119). In another study, we evaluated both B and T cell subsets on genetically unsolved CVID to show association of B and T lymphocyte abnormalities with the incidence of CVID. We indicated reduced number of total, naϊve, memory B cells and plasmablasts, decrease in number of total, naϊve, central memory and regulatory CD4^+^ T cells and naϊve CD8^+^ T cells as well as an increase in CD21^low^ and transitional B cells, effector memory and T_EMRA_ CD4^+^ T-cell subsets as well as total, effector memory, T_EMRA_, activated and cytotoxic CD8^+^ T cells on genetically unsolved CVID patients (120). Some novel correlations between lymphocyte abnormalities observed only among patients without monogenic defects and in the absence of any genetic defect may hint toward genetically unsolved CVID. Reduced levels of total and naive T cells and Tregs besides increased EM and T_EMRA_ T cells seem to have a direct impact on changes in B cell subsets, but activated and cytotoxic CD8^+^ T cells indirectly influence B cells alterations.

## Conclusion

Monogenic forms of CVID account for the pathogenesis of 20-50% of CVID patients. Based on the normal function of the mutated gene in the development of the B and T cells, monogenic CVID disorders display different immunophenotypes, including changes in switched memory B cells, NK cells, plasma cells, transitional B cells, follicular T helper cells, marginal zone B cells, etc. that may even vary in patients with the same mutation. The varied alteration of B- and T-cell subsets depends on the maturational arrest of the B- and T-cell development. Since there are only a few case report studies on different monogenic CVIDs, attribution of specific clinical manifestations and their frequency in the affected patients to a definite gene mutation requires more investigations.

Overall, recurrent respiratory infections, chronic diarrhea, splenomegaly, autoimmune diseases, thrombocytopenia (immune-related), lymphadenopathy, glomerulonephritis, etc., are common in patients with monogenic CVID. Having a comprehensive view of B- and T-cell subset alterations in monogenic CVID and using the laboratory data that is specific and almost distinct for most of the CVID patients with monogenic defects, the affected patients could be timely diagnosed, also targeted gene sequencing instead of whole-exome sequencing (WES) could be implemented for the molecular dissection. Thus, this approach will help for a rapid, cost-effective, time-saving diagnosis and is critically important for the optimized treatment required for improving the patients’ quality of life as well as accurate genetic counseling in the affected families.

## Author Contributions

SF, SK, and RY: substantial contributions to draft the article. HA and RY contributed to the design of the article or reviewed it critically and gave final approval of the version. All authors listed have made a substantial, direct, and intellectual contribution to the work and approved it for publication.

## Conflict of Interest

The authors declare that the research was conducted in the absence of any commercial or financial relationships that could be construed as a potential conflict of interest.

## Publisher’s Note

All claims expressed in this article are solely those of the authors and do not necessarily represent those of their affiliated organizations, or those of the publisher, the editors and the reviewers. Any product that may be evaluated in this article, or claim that may be made by its manufacturer, is not guaranteed or endorsed by the publisher.
